# Determination of total carbon dioxide in beer and soft drinks by gas diffusion and flow injection analysis

**DOI:** 10.1155/S1463924695000186

**Published:** 1995

**Authors:** Esbjörn Ljunggren, Bo Karlberg

**Affiliations:** Department of Analytical Chemistry Stockholm University Stockholm S-106 91 Sweden

## Abstract

A gas diffusion FIA method for determination of total CO_2_ in beer and in soft drinks is described. The composition of the acceptor stream for diffused carbon dioxide is critical. Bromocresol purple has been selecled among a large number of tested pH indicators and if the detection is made at a wavelength of 430 nm, stable baseline conditions and positive deflections of the resulting FIA peaks are obtained. The selected indicator is combined with a linear pH buffer. A simple and practical graphical method for determining a suitable starting pH of the acceptor stream, as well as the expected dynamic range and the linearity of the calibration graph, is presented.


Journal of Automatic Chemistry, Vol. 17, No. 3 (May–June 1995), pp. 105–108

### Determination of total carbon dioxide in beer and soft drinks by gas diffusion and flow injection analysis

**Esbjörn Ljunggren and Bo Karlberg**

*Department of Analytical Chemistry, Stockholm University, S-106 91 Stockholm, Sweden*

*A gas diffusion FIA method for determination of total $CO_2$ in beer and in soft drinks is described. The composition of the acceptor stream for diffused carbon dioxide is critical. Bromocresol purple has been selected among a large number of tested pH indicators and if the detection is made at a wavelength of 430 nm, stable baseline conditions and positive deflections of the resulting FIA peaks are obtained. The selected indicator is combined with a linear pH buffer. A simple and practical graphical method for determining a suitable starting pH of the acceptor stream, as well as the expected dynamic range and the linearity of the calibration graph, is presented.*

### Introduction

Determination of total carbon dioxide ($CO_2$) by gas diffusion FIA was first described by Baadenhuijsen and Seuren-Jacobs [1] for plasma samples. In this method, a silicone-rubber membrane in a gas diffusion cell and a combined carrier/acidic reagent stream was used into which 50 µl of the sample was injected. Cresol red was used as indicator and detection was performed at 410 nm. This basic FIA system has been developed further [2, 3] and theoretical considerations of the gas diffusion process have been presented [4, 5].

In a gas diffusion FIA system, the number of variables is large [6], so it is necessary to carry out screening in order to select significant variables. The response function determines the number and character of significant variables. Sensitivity has most frequently been used as a response function [7, 8]. For beer and soft drink samples, however, sensitivity is not a concern since these samples contain large amounts of total $CO_2$. Instead, the dynamic range becomes an important issue because the indicator can be totally consumed by the diffused gas. Consequently, a pH buffer must be added to the acceptor stream so that the major portion of the diffused gas reacts with the buffer, thereby enhancing the dynamic range.

For high concentrations of total $CO_2$ in the sample, a manifold, such as the one described by Kuban and Dasgupta [3], is preferred. In this approach, the sample is injected into a separate carrier stream and then merged with the acid reagent which is used to convert carbonate to gaseous carbon dioxide. The concentration of added acid across the entire sample zone is thus maintained at a constant value. This is in contrast to a single line arrangement where the added acid concentration attains a minimum value when the sample concentration is at its maximum value; this occurs in the centre of the sample plug. The acid concentration must be high enough to cause a sufficient decrease in sample pH so that $CO_2$ is quantitatively formed. A pH value below 4 is required, i.e. at least 2 $pK_a$ units below the first dissociation constant of the protonized acid. The choice of pH indicator for the acceptor stream influences the sensitivity and the baseline stability. Considering baseline stability alone, a low absorbance value is preferred when the system is in a stand-by mode. Thus, an increase in the absorbance is obtained when samples containing carbonate are injected.

This paper describes a procedure and a gas diffusion FIA system for determination of total $CO_2$ in beer and soft drinks. A simple evaluation method for different acceptor solutions with respect to expected dynamic range and expected linearity of the calibration graph, as well as results from runs of real samples, is presented.

### Experimental

### *Instruments and apparatus*

A commercial flow injection system (FIAstar 5020 and 5023, Tecator) was used. The gas diffusion cell (Chemifold V, Tecator) was provided with a PTFE gas diffusion membrane (homogeneous plumber's tape); the FIA manifold is shown in figure 1. The sample volume was 100 µl. The mixing coil length was 30 cm, and the inner diameter was 0·7 mm.

Indicator screening experiments entailed collection of wavelength spectra for various indicator solutions in the range 350–800 nm by means of a diode array spectrophotometer (Hewlett-Packard 8452A).

### *Reagents, solutions and samples*

Deionized water was used as carrier and reagent R1 consisted of 0.2 M sulphuric acid. Reagent R2 was the pH indicator solution: bromocresol purple. A stock

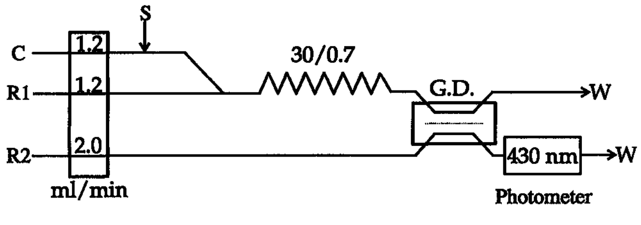

*Figure 1. FIA manifold for determination of total $CO_2$. S = sample; C = carrier; R1 = $H_2SO_4$; R2 = indicator (acceptor) solution; and G.D. = gas diffusion cell.*

0142-0453/95 $10.00 © 1995 Taylor & Francis Ltd.

*Table 1. Screening results for all indicators investigated.*

| Indicator | Concentration (g/l) | Isobestic point (nm) | Suitable wavelength (nm) | Corresponding absorbance (A.U.) | |
|---|---|---|---|---|---|
| | | | | pH 4 | pH 9 |
| Bromophenol blue* | 0·07 | — | 440 | 1·00 | 0·16 |
| Bromocresol green | 0·028 | 510 | 440 | 0·70 | 0·15 |
| Bromocresol purple | 0·027 | 490 | 430 | 1·00 | 0·10 |
| Cresol red | 0·033 | 485 | 430 | 1·90 | 0·40 |

*This indicator had complex spectra.

solution was prepared by dissolving 0·1 g of bromocresol purple in 18·5 ml 0·01 M NaOH and adding water to a final volume of 250 ml. R2 is prepared by taking a 10 ml portion of the indicator stock solution and mixing with 1 ml of a linear buffer, which was prepared according to Åström [9]. This buffer consisted of formic acid (0·0225 M), acetic acid (0·0150 M), malonic acid (0·012 M), piperazine (0·0218 M), Bis-tris (0·0144 M), *N*-methylmorpholine (0·0226 M), *N*-methyldiethanolamine (0·0223 M), and sodium chloride (1.0 M) and was pH-adjusted by addition of NaOH which corresponded to a concentration of 0·034 M NaOH in the final solution. Carbonate standard solutions were prepared from sodium hydrogen carbonate. Deionized water was used throughout and all chemicals were of reagent grade. The samples were all chilled at +4°C.

When the wavelength screening of different potential indicators for R2 was performed, the following pH buffers were used: pH 4–0·05 M KH-phthalate; pH 7–0·025 M phosphate; pH 9–0.01 M borax. A stock solution of each pH indicator was prepared by taking a certain amount of the indicator and then diluting it 1:15 with addition of the various buffer solutions. Final concentrations for four of the indicators are given in table 1.

*Designing the FIA system*

Three variables in the FIA system shown in figure 1 were subjected to a two-level reduced factorial design, namely the sulphuric acid concentration of R1 (0·2/2·0 M), the coil length (30/60 cm), and the coil inner diameter (0·5/0·7 mm). All other variables were kept constant. None of the three studied variables was found to be significant when the peak height and the repeatability were employed as responses. The variable values selected are shown in figure 1.

### Results and discussion

*Screening of indicators*

More than 20 indicators were tested for possible use when composing reagent R2. Spectra were recorded for each indicator at three different pH values: 4, 7, and 9. In figure 2 a typical set of spectra is shown for bromocresol purple. For most indicators an isobestic point is found. On either side of this isobestic point, absorbance maxima appear. At these maxima, the absorbance is either

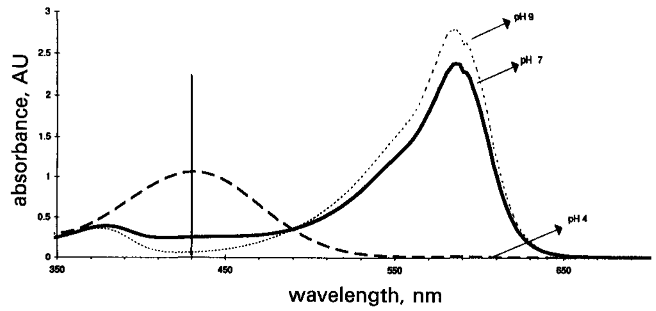

*Figure 2. A typical set of spectra obtained when screening indicators at different pH values; this set of spectra was obtained for bromocresol purple.*

increasing or decreasing as a function of pH. Because carbon dioxide diffusing through the membrane and entering the acceptor stream R2 will cause a decrease in pH, it is desirable that this decrease in pH will result in a corresponding increase in indicator absorbance. This is fulfilled in the 390–470 nm region for bromocresol purple, see figure 2. Thus, a positive deflection of the FIA response curve is registered in this wavelength range. A maximum deflection is found at the absorbance maximum attained by the pH 4 curve, see the vertical full line at 430 nm in figure 2.

The indicators listed in table 1 could be used for composing the reagent R2. However, the choice is dictated by the additional requirement that the background absorbance of the indicator at a starting pH value of 8–9 must be low. If the starting absorbance is too high, the validity of Beer's law might be jeopardized, resulting in non-linear calibration graphs at high analyte concentrations. Bromocresol purple was finally selected because a stable and reliable baseline appeared at 430 nm, in contrast to the baselines obtained for bromocresol green and cresol red. Bromophenol blue might be the second-best choice.

*Composing the acceptor solution (R2)*

The shape of the calibration graph is governed by the starting pH value and the buffer capacity of the acceptor solution, R2. Figure 3 shows the results of a simple evaluation method for an acceptor solution consisting of bromocresol purple and linear buffer. The starting pH of the acceptor solution was 12. Titration with hydrochloric acid and simultaneous recording of the absorbance values at 430 nm were then performed. The results in figure 3 provide useful information regarding a

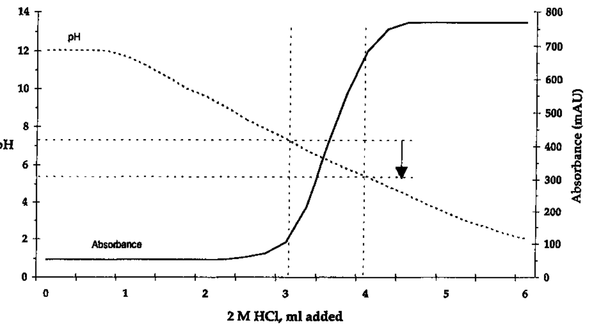

*Figure 3. Changes in pH and absorbance of an acceptor solution (bromocresol purple indicator and linear pH buffer) after addition of hydrochloric acid.*

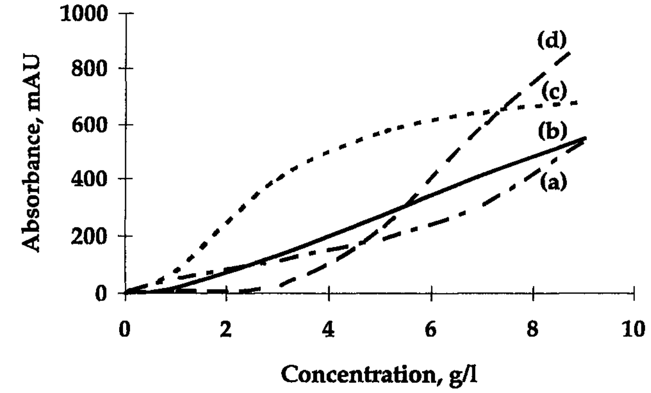

*Figure 4. Four different types of calibration graphs for determination of total $CO_2$. For explanation, see text.*

suitable starting pH value of the acceptor solution, the dynamic range and, finally, the expected linearity of the calibration graph.

In figure 4, four different types of calibration graphs are presented. The shape of each one of these can be explained by referring to figure 3. For curve (a) in figure 4, a starting pH value which was too high was selected; for curve (b), it appears that appropriate conditions were selected. For curve (c), a starting pH value which was too low was selected. For curve (d), a 'zero suppression' was accomplished by starting at a high pH value; which results in poor sensitivity at low concentrations of carbonate.

### *Real samples*

Results for a limited selection of beer and soft drinks with the FIA system shown in figure 1, using bromocresol purple and a linear buffer as an acceptor stream for the diffused carbon dioxide, are shown in table 2. The numbers of respective replicate runs are denoted by 'n' in table 2. Standard deviation values are given in the same table. The reported analytical results are in full agreement with results obtained by using the commonly applied titration method.

As expected, the sample pretreatment procedure is crucial. When opening a bottle or a can, carbon dioxide evolves and will be lost if the partial pressure of the gas is not substantially lowered. A direct detection of carbon dioxide

*Table 2. Determination of total $CO_2$ in real samples.*

| Sample, chilled at +4°C | Pretreatment* | Concentration ±s.d. (g/l) | n |
|---|---|---|---|
| Tuborg beer, 2·8% alc. by weight | 1 | 5·1 ± 0·05 | 8 |
| | 2 | 3·9 ± 0·10 | 10 |
| | 3 | 3·6 ± 0·02 | 5 |
| | 4 | 3·4 ± 0·01 | 5 |
| | 5 | 3·3 ± 0·04 | 7 |
| | 6 | 5·2 ± 0·09 | 8 |
| Pripps beer, 1·8% | 1 | 4·9 ± 0·05 | 3 |
| Coke | 1 | 5·4 ± 0·01 | 3 |
| | 2 | 4·6 ± 0·10 | 3 |
| | 3 | 3·5 ± 0·29 | 5 |
| Coke, diet | 1 | 6·3 ± 0·10 | 3 |
| Club soda** | 1 | 8·0 ± 0·02 | 3 |

*1: Prompt addition of 15 ml 10 M NaOH/l after opening the bottle.
2: The bottle was opened; contents transferred to a beaker; addition of 15 ml 10 M NaOH/l.
3: The bottle was opened; contents transferred to a beaker; allowed to stand for 5 min; addition of 15 ml 10 M NaOH/l.
4: The bottle was opened; contents transferred to a beaker; treatment in ultrasonic bath for 2 min; addition of 15 ml 10 M NaOH/l.
5: Prompt addition of 20 ml triethanol amine/l.
6: Prompt addition of 15 ml 10 M NaOH/l and 20 ml triethanol amine/l.
**The chilling procedure of this sample was apparently not sufficient, bubbling occurred when opened.

in the original sample by, for instance, a $CO_2$ electrode is, for obvious reasons, impossible. However, by increasing the pH of the sample by adding sodium hydroxide, volatile carbon dioxide is transferred to carbonate and determination of total $CO_2$ in the original sample can be accomplished. Triethanolamine, in combination with sodium hydroxide, is suitable for adding to the sample, but the exact procedure for this addition must be carefully defined to guarantee reproducible results.

### Conclusions

When large concentrations of analyte, such as carbonate, sulphite, and ammonium, are to be determined by gas diffusion in an FIA system, the composition of the acceptor stream is critical. The acid-base indicators usually employed cannot be added to the acceptor stream in sufficient quantities to produce the buffering capacity needed for a wide dynamic range. A separate buffer is required. Theoretically, the dynamic range can be calculated for any given composition of the acceptor stream using $pK_a$ values. In practice, however, an evaluation method such as the one described and illustrated in figure 3 is to be preferred.

### Acknowledgement

The authors are indebted to Robert Tryzell for valuable discussions.

### References

1. Baadenhuijsen, H. and Seuren-Jacobs, H. E. H., *Clinical Chemistry*, 25 (1979), 443.

2. Motonizu, S., Toei, K., Kuwaki, T. and Oshima, M., *Analytical Chemistry*, **59** (1987), 2930.

3. Kuban, V. and Dasgupta, P. K., *Talanta*, **40** (1993), 831.

4. Van der Linden, W. E., *Analytica Chimica Acta*, **151** (1983), 359.

5. Van der Linden, W. E., *Analytica Chimica Acta*, **155** (1983), 273.

6. Tryzell, R. and Karlberg, B., *Analytica Chimica Acta* (in press).

7. Nakata, R., Kawamura, T., Sakashita, H. and Nitta, A., *Analytica Chimica Acta*, **208** (1988), 81.

8. Risinger, L., Johansson, G. and Thorneman, T., *Analytica Chimica Acta*, **224** (1989), 13.

9. Åström, O., *Analytica Chimica Acta*, **97** (1978), 259.

